# Circular design strategies and economic sustainability of construction projects in china: the mediating role of organizational culture

**DOI:** 10.1038/s41598-024-56452-0

**Published:** 2024-04-03

**Authors:** You Chen, Xiaomin Yin, Chunwei Lyu

**Affiliations:** 1 Department of Architecture, Wuhan University, Wuhan, 430072 China; 2 School of Marxism, Zhejiang Shuren University, Hangzhou, 310015 Zhejiang China; 3 School of Educational Studies, Universiti Sains Malaysia, 11700 Gelugor, Penang Malaysia

**Keywords:** Circular design, Strategies, Organizational culture, Construction projects, Economic sustainability, Energy science and technology, Engineering

## Abstract

This research aims to elucidate the relationship between circular design strategies (CDS) and the economic sustainability of construction projects (ESCP), examining the mediating role of organizational culture (OC). Motivated by the imperative to develop a sustainable circular economy (CE) model in the building industry, our study focuses on a crucial dimension of CE processes. Specifically, we investigate how construction firms’ organizational values shape their pursuit of desired economic outcomes within CE theory. Through a comprehensive analysis of 359 responses from a cross-sectional survey of Chinese construction firms employing Partial Least Squares-Structural Equation Modeling (PLS-SEM), our findings reveal a positive albeit weakly impactful association between CDS and ESCP. Simultaneously, OC is identified as a factor detrimental to ESCP. Notably, this study unveils the influential roles of hierarchical culture (HC) and group culture (GC) in shaping the current state of ESCP in China. Emphasizing the significance of CDS, we propose that contract administrators proactively reposition their organizations to adopt strategies conducive to achieving the necessary economic output for construction projects. The originality aspect lies in this research contributes to the existing body of knowledge by offering empirical insights into the theoretical framework, marking the first such empirical study in northern China. We conclude by critically examining research outcomes and limitations while providing insightful recommendations for future research to foster sustainable construction practices in the Chinese context.

## Introduction

Current and upcoming trends point to the necessity for a fundamental shift in how resources are used to avoid ecological breakdown and significant interruptions in production^1^. The rate at which the human population grows, accompanied by a growth in the population’s purchasing power, always results in the depletion of additional material resources. Yin et al^2^. discussed how the world’s population is quickly urbanizing, with a 70% urbanization rate expected by 2050. China, one of the fastest-urbanizing countries in Asia, today has nearly half its population living in urban areas^3^, signalling the need for more infrastructure^4^. Linear material use is depleting scarce resources and accumulating global waste problems^5^. Given the costs of extracting, refining, and creating materials, maximizing the material’s value is critical to ensuring that it is kept in circulation (in terms of function and service) for as long as possible. A material flow paradigm, in which the flows are inverse, is more likely to solve the dilemma of an unsustainable universal linear flow economy^6^. The idea of the circular economy (CE) is a growing method that is increasingly frequently explored to overcome the existing quandary of product lifecycles to achieve a more sustainable, workable economic structure. The already prevalent topic of sustainable development, which has captivated worldwide consciousness and execution, bolsters the circularity mentality^7^. Transitioning to a working CE rule necessitates a multi-level transformation that includes technical modernization, new business models, and, most crucially, unwavering stakeholder cooperation^8^.

Policymakers and other stakeholders have recently expressed an interest in developing a circular economy model in the building industry^9^. According to the UK Green Building Council (UKGBC, 2019), CE must be viewed as a commercial strategy, not only a sustainability consideration, if economic opportunities are to be achieved. The building sector consumes 40% of processed timber globally and accounts for 16% of total water energy, yielding 40% of all raw materials and 25% of all resources mined in industrialized countries^10,11^. This vital figure highlights the extent to which the building industry is involved in the environment. Construction materials are becoming increasingly scarce in many parts of the world. Shifting to CE and other sustainability-driven business models necessitates a significant transformation that affects the entire construction company and its employees. This shift necessitates creative solutions that replace present systems with ones that are more circular in nature. To identify strategies for organizations to regulate this disruptive transformation, it is necessary to start from within the organization to understand the challenges and barriers they face^12^. Firms’ initiatives to shift to CE are insufficient^9,13^. A CE’s essential concept must reach every level of any organization wanting to operate according to its standards for it to be fully implemented^14^. The globally recognized idea of Organizational Culture (OC) of these organizations is at the heart of this argument. The impact of OC on a company’s operations and production has been recognized in the literature for a while now^15–21^. Prior research has shown OC as a background element or social context that impacts a firm’s learning processes in acquiring and applying specialized knowledge^22^.

The construction industry is a pivotal sector in the global economy, especially in rapidly urbanizing nations like China^23,24^. As the industry evolves, an increasing emphasis is on integrating sustainable practices to mitigate its environmental impact and promote economic viability. This study delves into the nuanced interplay between Circular Design Strategies (CDS) and the Economic Sustainability of Construction Projects (ESCP)^9,25^, with a particular focus on the mediating role of OC in this dynamic. The concept of a circular economy, characterized by reduced waste and continual use of resources, offers a promising pathway to sustainability in the construction sector^22,26^. However, the transition to a circular economy involves adopting innovative design strategies and a fundamental shift in the organizational culture within construction firms^19,27^.

The primary objective of this research is to unravel the complexities of implementing Circular Design Strategies in the construction industry and their impact on the economic sustainability of projects. We aim to understand how organizational culture influences this relationship, potentially acting as a catalyst or a barrier. By examining the specific context of the Chinese construction industry, this research seeks to contribute valuable insights into how firms can navigate the transition towards a circular economy, highlighting the critical role of organizational values and practices in this transformative journey.

Specifically, the research questions are refined and consolidated into a cohesive inquiry: How do Circular Design Strategies influence the Economic Sustainability of Construction Projects in the Chinese construction industry, and to what extent does Organizational Culture mediate this relationship? This question encapsulates the essence of our investigation, aiming to provide a holistic understanding of the interdependencies between design strategies, economic sustainability, and the underlying organizational ethos within the construction industry in China.

## Literature review

### Organizational culture in China

Organizational culture has been defined in various ways, capturing its essence as a set of shared values, beliefs, and assumptions within an organization^2,15^, which defined as a set of “values, beliefs and salient assumptions that members of organizations have in common”^3,16–18,28,29^. The earliest specific definition of OC is “a pattern of basic assumptions—invented, discovered, or developed by a given group as it learns to cope with its problems of external adoption and internal integration—that has worked well enough to be considered valid and, therefore, to be taught to new members as the correct way to perceive, think, and feel concerning those problems”^30^.

Organizational culture shapes an organization’s behaviour, practices, and outcomes. Examining corporate culture in China is particularly insightful given its unique historical, social, and economic context, deeply rooted in its rich history, Confucian philosophy, and rapid socio-economic transformations. While the general definition of organizational culture applies, the Chinese context adds cultural nuances, emphasizing collectivism, hierarchy, and the significance of relationships^15,16^. Understanding guanxi, the intricate network of personal connections is crucial for grasping the intricacies of organizational dynamics in China^2,16^.

The dimensions of organizational culture in China reflect a blend of traditional values and modern influences^4,7,25^. Collectivism, harmony, and a strong sense of social hierarchy are prominent cultural dimensions, aligning with Confucian principles^17–19^. The influence of these dimensions can be observed in workplace dynamics, decision-making processes, and the emphasis on group cohesion over individual achievement.

Measuring organizational culture in China requires culturally sensitive instruments. The Hofstede Cultural Dimensions Model, with dimensions such as power distance, individualism-collectivism, and uncertainty avoidance, has been adapted to assess organizational culture in the Chinese context^20,21^. Additionally, instruments like the Chinese Organizational Culture Assessment Instrument (COCAI) have been developed to capture the unique aspects of organizational culture in Chinese enterprises^15,16,19^.

Organizational culture significantly influences employee behaviour in Chinese organizations. The emphasis on harmony and group cohesion fosters a collaborative and team-oriented work environment^3,15,21^. Leadership is crucial, with leaders often embodying Confucian values of benevolence and moral integrity^9^. This cultural backdrop influences Chinese organizations’ communication styles, decision-making processes, and conflict-resolution approaches. That’s the justification for this research selected this aspect to shed light on the mediating role among circular design strategies and economic sustainability of construction projects.

The relationship between organizational culture and performance in China is complex. The emphasis on collective goals can enhance teamwork and employee engagement, positively impacting organizational outcomes^3,17,18,21^. However, challenges arise when traditional cultural values clash with the demands of a rapidly evolving business landscape, requiring organizations to navigate a delicate balance between tradition and modernity^7,31^.

Their unique cultural context influences innovation within Chinese organizations. While traditional values may emphasize stability and conformity, there is a growing recognition of the need for innovative thinking^32–36^. Companies in China are increasingly adopting a more flexible and open organizational culture to encourage creativity and adaptability^33–35^.

Navigating change within Chinese organizations requires understanding the cultural dynamics at play. The cultural preference for stability and respect for authority may challenge rapid organizational transformation^34^. Successful change initiatives often involve aligning corporate culture with innovation and global competitiveness goals, requiring leaders to manage cultural shifts^7,31,34^.

Generally, every existing organization has its distinct cultural characteristics. In this context, culture refers to how groups, including companies, interact internally and externally. As a result, the cultural coherence of a specific organization is determined by the sufficiency of the group’s artistic mission, strategies, and processes^37^. These cultural techniques are referred to as “macro dimensions,” which are characterized by the dynamic nature of organizations, in which numerous forces interact and impact one another and are thus sensitive to changes that may alter the structure of the organization^37^.

To effectively empirically analyze the effects of OC in line with the stated objectives, this study adopts the Competing Values Framework (CVF) model advanced by Quinn and his co-researchers in the eighties^38–41^. The CVF is the most recurrently engaged tool in theoretical and experimental studies relating OC to organizational performance^19^ and, hence, the most suitable to address CE-related activities. The CVF reveals the multifaceted compositions of OC specifically relating to submission, drive, management, choices and efficacy in an organization^15^.

Additionally, in light of variables that affect Circular Design Strategies (CDS) and Economic Sustainability of Construction Projects in China in the literature review, consider including variables like technological innovation, government regulations, market demand for green building, and supply chain practices. These factors significantly impact the adoption and effectiveness of CDS in contributing to economic sustainability.

In terms of justification for selecting organizational culture as a research variable, it fundamentally shapes how strategies are interpreted, implemented, and integrated within a company’s operations^19,38,41^. The Competing Values Framework (CVF) provides a multidimensional model to assess organizational culture, making it a robust tool for understanding the cultural underpinnings that influence CDS adoption^39^. Organizational culture affects every aspect of CDS implementation, from decision-making processes to employee engagement and resource allocation. This focus is particularly relevant in the Chinese construction industry, where rapid development and unique cultural factors necessitate a deep understanding of how organizational culture can facilitate or hinder the transition to more sustainable and economically viable construction practices.

### Circular design strategies

With the building industry consuming a significant quantity of finite resources, it is critical to use an appropriate structural strategy from the start (design), paying attention to the circularity and reusability of the different materials and components within it. Furthermore, it is critical to concentrate on the packaging that these components are wrapped in; this circularity-by-design approach is essential to creating a zero-waste circular economy in both production and consumption. If the construction industry is an example of how crucial steps of circularity can be achieved through design, then it is critical to plan for reuse and recovery^42,43^. This refers to determining if an existing structure could be entirely or partially reused and incorporated into the new design. Materials, components, packaging, and other items that can be reused, remanufactured, returned, or repurposed would be included in the design^44^. Design for flexibility and deconstruction is a vital feature here, with the goal of creating a process and a structured system that decreases the life of waste while simultaneously designing a model for reuse and repurposing. The flexibility option in design is a significant tool for promoting the adoption of a CE system in various businesses and sectors^45^. On the one hand, the sustainability and Circular Economy (CE) paradigms^46^ enhanced awareness of natural resource scarcity among customers and providers. Manufacturers have been forced to change their business strategies to pursue this transformation^47^ by detecting and avoiding associated^48^ and exploring potential advantages^43^.

### Economic sustainability of construction projects

Practices supporting long-term economic growth without negatively harming the community’s social, environmental, or cultural components are called “Economic Sustainability (ES)”^49^. Financial aspects in construction refer to all costs and benefits associated with construction-related functions, from the initial capital investment to operating gains and final return proceeds^23,50^. Clients and owners naturally prioritize these issues, especially when breakthroughs or technology are introduced into the sector. Apart from that, how CE adoption leads to ES, which reduces building lifespan maintenance costs, is also demonstrated^51^. They stated that ES is guaranteed when circular design techniques are used, as they promise profitability without compromising people’s demands. Standardization of circular design and construction approaches reduces costs by minimizing repeats while saving on long-term operational costs due to fewer repairs and replacements^52^. They also discovered that ES is reached due to the ease with which materials may be retrieved, lowering material End-of-Life (EoL) costs. ES is thought to be attained when a client receives a good return on their investment by using CE processes and maintaining the quality of the end goods using modular building approaches that reduce waste and increase productivity. Another point of view is that the local economy is bolstered by the development of jobs in recycling and repair procedures, assuring the economy’s long-term viability^53^. Overall, the client’s economy improves due to the lower cost of acquiring new materials, a more sophisticated and consistent product quality and reliable performance achieved through repetitive design and production of modular components^54^.

### Hypothesis development

#### Circular design strategies and organisational culture

**Figure 1:**
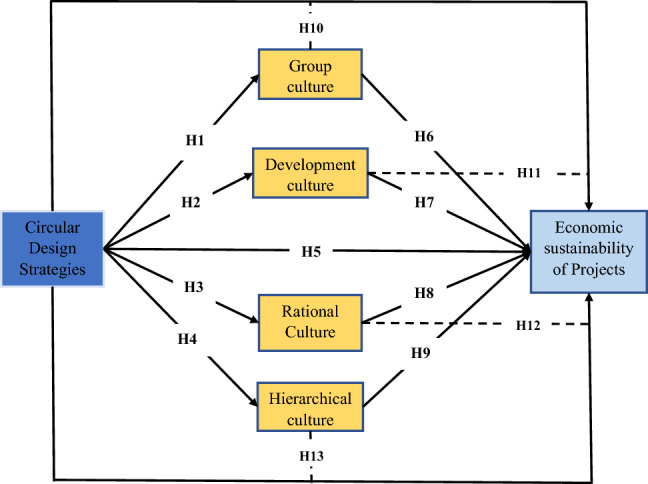
Research conceptual and hypothetical framework.

As more businesses employ design thinking techniques to address organizational problems, the need to establish corporate cultures that support the effective use of these tools may become more significant^55^. At the same time, past research has shown that cultural changes usually happen in stages due to organizational life cycle elements, demographic shifts, and members’ exposure to broader societal or professional culture shifts^56^. When rapid changes in corporate values and assumptions are required (for example, in the case of firms adopting circular design strategies), the role of organizational leaders, supported by company-wide initiatives (for example, training, coaching, and role modelling), may be critical in overcoming corporate culture’s natural inertia^57^. Construction companies must build a culture that nurtures creativity and promotes innovation to succeed and “remain externally flexible.” Ismail^58^ claims that “innovation will be fostered when culture and way of thinking mix to create new ideas”^59^. Employees will be motivated to take an active role in decision-making and share their innovative ideas with management to improve organizational performance if the company has a strong culture^60^. As such, the following hypotheses were formulated to examine the extent of the relationship between the independent variable (circular design strategies) and the mediating variable (the CVF of group, developmental, rational, and hierarchical cultures). For this section, four hypotheses were developed for the relationship between the IV and each of the dimensions of the MV. The hypotheses were grown, therefore, to assess the relationship based on the proposed research framework shown (Fig. 1).

##### *H1: *

*Circular Design Strategies have a significant positive effect on Group Culture.*

##### *H2: *

*Circular Design Strategies have a significant positive effect on Development Culture.*

##### *H3: *

*Circular Design Strategies have a significant positive effect on Rational Culture.*

##### *H4: *

*Circular Design Strategies have a significant positive effect on Hierarchical Culture.*

#### Circular design strategies and economic sustainability

Sustainable and flexible ideas are crucial characteristics of a good building design^57^; architects are expected to design robust and long-lasting structures; otherwise, they are fundamentally untenable. The idea is to encourage architects to consider designs for reducing waste generation, as considering waste minimization measures before generating waste is less expensive. For instance, modular construction and design can reduce waste generation on construction sites because fabrication is carried out at factories where production is controllable, thereby improving the economic sustainability of construction^36^. Additionally, empirical evidence that aids in evaluating sustainable methods will tremendously assist clients and can pave the way for future sustainable products^61^. Hence, the following hypothesis was formulated to examine the extent of the relationship between the independent variable (circular design strategies) and the dependent variable (the Economic sustainability of construction projects). The hypothesis was developed, therefore, to assess the relationship based on the proposed research framework shown (Fig. 1).

##### *H5: *

*Circular Design Strategies have a significant positive effect on Economic sustainability.*

#### Organisational culture and economic sustainability

According to Isensee et al^62^**.,** firms that do well in sustainability have a distinct organizational culture. Construction firms are also part of the business sector, and focusing on sustainability can help them compete more effectively. Offering sustainable products and services allows segmentation and customization to satisfy specific needs^63^. There appears to be no single approach to explain how innovations will develop or how culture can be maintained in a building industry with such a diversified and multileveled nature. To be competitive, businesses must continuously develop fresh ideas to improve operations and become more inventive. This is critical for industries like construction as they migrate to more sustainable practices^64^.

Consequently, the following hypotheses were formulated to examine the extent of the relationship between the four dimensions of the MV (group, development, rational and hierarchical cultures) and the DV (ES of construction projects). For this section, four hypotheses were developed for the relationship between the dimensions of the MV (CVF of OC) and the DV (ES of construction projects). The hypotheses were grown, therefore, to assess the relationship based on the proposed research framework shown (Fig. 1).

##### *H6: *

*Group Culture has a significant favourable influence on Economic Sustainability*

##### *H7: *

*Development Culture has a significant favourable influence on Economic Sustainability*

##### *H8: *

*Rational Culture has a significant favourable influence on Economic Sustainability*

##### *H9: *

*Hierarchical Culture has a significant favourable influence on Economic Sustainability*

#### The mediating role of OC between CDS and ES

Based on those mentioned earlier, the following hypotheses were also formulated to examine the mediating effect of the four dimensions of the MV (group, development, rational and hierarchical cultures) between the IV (CDS) and the DV (ES of construction projects) in line with RQ4. For this section, four hypotheses were developed to assess the mediating relationship based on the proposed research framework shown (Fig. 1).

##### *H10: *

*Group Culture mediates the relationship between CDS and Economic Sustainability of projects*

##### *H11: *

*Development Culture mediates the relationship between CDS and Economic Sustainability of projects*

##### *H12: *

*Rational Culture mediates the relationship between CDS and Economic Sustainability of projects*

##### *H13: *

*Hierarchical Culture mediates the relationship between CDS and Economic Sustainability of projects*

### Theoretical background

The circular economy idea underpins this research. The CE was first introduced in the 1970s by the Swiss architect and economist Walter Stahel, who recommended that materials be managed in a ‘closed loop’, thereby turning waste into resources. Stahel defined this as a ‘Cradle-to-Cradle’ system and the Linear model as Cradle-to-Grave^65^. The need to stretch out product life through restoration and remanufacture was also emphasized^66–69^. Consequently, the Cradle to Cradle method was designed by William McDonough (architect) and Michael Braungart (environmental chemist), who stated that it would facilitate ‘design for abundance’^70^ as a result of which they developed the Cradle-2-Cradle (C2C) benchmark to approve and endorse products that justify such standards^44^. CE has evolved and continues to gain traction^71^. The mutual instituting ideologies are based on more outstanding resource organization and waste minimization^71–73^.

## Research framework

The current study is unique in that it examines the link between CDS and ESCP mediated by the competing values framework of OC governed by the Circular Economy theory. Figure 1 illustrates the hypothetical research framework proposed for the study.

## Methodology

Statement: This study confirms that all methods were carried out per relevant guidelines and regulations. Wuhan University’s institutional and licensing committee approved all experimental protocols. Informed consent was obtained from all subjects and legal guardian(s).

### Survey and data collection

For this study, the Positivist approach and Quantitative techniques were adopted as philosophy and design, respectively, to ascertain the mediating effects of OC on the relationship between CDS and ESCP in the Chinese construction industry. This is to effectively analyze the causal relations and demonstrate the level of direct and indirect relationships among all variables. Given the study’s predefined philosophy and design, a survey technique was used to satisfy the stated goals. As a result of the background and scope provided, the target population for this study was all contracting and consulting firms in the study area. Only northern China was considered for this study, and the reasons were not far-fetched. So far, the southern region of China has hosted many studies on CE implementation and processes^7,31–34^, leaving no clear indices on the subject matter for the northern part. The north area currently consists of three geopolitical zones: North-west, North-Central and North-East. Two provinces from each zone will be picked for the study for appropriate spread, with Shanxi and Qinghai Province representing the North-west, the Inner Mongolia and Hebei provinces representing the North-central zone and Heilongjiang and Jilin Province representing the North-east. These provinces were carefully chosen since they represent areas with a high tendency for construction-related activities.

The total research population is **1535** obtained from https://libguides.library.cityu.edu.hk/busChina/comp-dir (an online directory of registered firms in China, December 2023 version) because of a dearth of data on available construction entities in the industry. However, the population for the selected province was found to be **778**. This was rounded to **800** to attain a research sample **260** based on the Shanxi and Qinghai Province 1970 rule of thumb^74^. The study adopted the proportionate stratified random sampling technique for its suitability in ensuring adequate representation of respondent elements^75^. The data was collected for six months, from June 2019 until November 2023. E-mails and WeChat messages attached with an online link for Google Forms were sent to respondents to complete. Follow-up calls and gentle reminders were sent to research assistants via phone for quick responses and full cooperation. The questionnaires were accompanied by cover letters that included a brief description of the research and assurance of anonymity and confidentiality. In total, 550 survey questionnaires were distributed to Construction Contracting and Consulting Companies in Northern China, of which 390 were returned. Out of the returned questionnaires, 359 were used for data analysis. Data were obtained from Principal partners, Partners, Management staff, Project Managers and Regular staff of these organizations through the survey instruments. Table 1 highlights the statistics of questionnaires distributed, including return and response rates.

**Table 1:** Questionnaire distribution, return and response rate.

Region	Province	Respondents	Sample required	Questionnaire distributed	Questionnaire returned	Questionnaire missing	Used for analysis	Return rate (%)	Response rate (%)
North Central	Inner Mongolia	Contractors	62	100	87	13	81	87	81
		Consultants	53	100	76	24	73	76	73
	Hebei	Contractors	18	35	31	4	28	89	80
		Consultants	12	30	17	13	16	57	53
North West	Shanxi	Contractors	26	50	38	12	35	76	70
		Consultants	18	40	30	10	26	75	65
	Qinghai	Contractors	15	35	20	15	18	57	51
		Consultants	14	30	23	7	21	77	70
North East	Heilongjiang	Contractors	10	35	18	17	16	51	46
		Consultants	8	30	12	18	11	40	37
	Jilin	Contractors	14	35	23	12	22	66	63
		Consultants	10	30	15	15	12	50	40
Total			**260**	**550**	**390**	**160**	**359**	**71%**	**65%**

Significant values are in [bold].

### Measurements

The measurement for Design Strategies (9 items), Competing values framework of organizational culture (22 items) and Economic sustainability of construction projects (10 items) were adopted from the sources highlighted in Table 2. A 5-point Likert scale was employed for the use of this study. The Likert scale for Design strategies adopted ranged from “very low”, denoted by 1, to “very high”, represented by 5. The Likert scale for Organizational culture ranged from “strongly disagree”, characterized by 1, to “strongly agree”, denoted by 5. The Likert scale for Economic sustainability indicators ranged from “very low”, indicated by 1, to “very high”, marked by. Additionally, the respondents were provided with descriptions for every construct, with precise directions on completing the assessment of the items to prevent any confusion. Table 2 demonstrates the constructs and variables used in this study.

**Table 2:** Questionnaire constructs, items and sources.

Construct/dimensions		Items	Sources
Circular design	Design strategies	9	^42–45,76,77^
CVF of OC	Group culture	5	^15,18^
	Development culture	6	
	Rational culture	6	
	Hierarchical culture	5	
ES of construction projects (DV)	ES of construction projects	10	^51–54^

## Results

### Demographic profile

The demographic profile shows that respondents possessed adequate experience, education, and the correct position to respond to this study. Project managers accounted for 30% of the respondents, with the remaining percentage taken by Administrative staff (26%), Partners (10%), Principal staff (8%) and Project support staff (27%). Regarding professional background, Architects, Quantity Surveyors and Builders they have accounted for 85% of the respondents. About 62% of the respondent organizations (Contracting = 57%, Consulting = 43%) have operated for years ranging between 15 years and above. Regarding educational background, 60% of respondents possess a minimum master’s degree, and 38% have a bachelor’s degree. 86% of responding firms engage in Building projects, while 14% engage in Engineering projects.

In total, 550 survey questionnaires were distributed to Construction Contracting and Consulting Companies in Northern China, of which 390 were returned. For the returned questionnaires, 359 were deemed usable after being filtered for errors, incompleteness, or missing data, thus giving a response rate of 65%. Thirteen hypotheses were formulated and tested in this study. Data were obtained from Principal partners, Partners, Management staff, Project Managers and Regular staff of these organizations through survey questionnaires.

### Data analysis

The PLS-SEM technique using SmartPLS 3.3.3^78^ software was selected to examine the research model. PLS can handle complex structural equation models with many constructs and non-normal data distribution. Hence, this approach was used^79^. Table 3 portrays Mardia’s multivariate skewness (_β = 7.612, *p* < 0.01) and kurtosis (β = 54.349, *p* < 0.01). If the b value for multivariate skewness is more significant than ± 3 and the b value for multivariate kurtosis is more critical than ± 20, then the data distribution is not normal^80^. As a result, PLS-SEM was used as recommended by^81^.

**Table 3:** Multivariate skewness and kurtosis.

	b	Z	*p*-Value
Skewness	7.612	455.469	< 0.01
Kurtosis	54.349	6.139	< 0.01

#### Common method variance

Because self-report questionnaires gathered the data for this study, there is a risk of statistical and methodological biases developing because the data came from a single source and had similar scale qualities^82^. A comprehensive collinearity test is one of the methods used to detect this issue. If two or more variables measure the same attribute of an object or construct, they are said to be collinear^83^. To calculate the Variance Inflation Factor (VIF) values, linear regression analysis was used in SPSS Statistic 23 software with a random variable as the dependent variable and other latent variables as independent variables. The value of 3.3 was chosen^83^ as the cut-off point for the VIF readings. CMV was not an issue and is unlikely to produce any problems for the current research findings because the VIF values (refer to Table 4) are below the threshold values.

**Table 4:** Collinearity.

Variable	CDS	DC	ESCP	GC	HC	RC
VIF	1.965	3.011	1.132	2.069	1.218	2.050

Note: CDS = Circular Design Strategies, DC = Development Culture, ESCP = Economic sustainability of Construction Projects, GC = Group Culture, HC = Hierarchical Culture, RC = Rational Culture.

#### Measurement model

The measurement model for this study was examined by checking the convergent and discriminant validity of the research data. The degree to which a measure correlates positively with other measures of the same construct is known as concurrent validity. The loadings, average variance extracted (AVE), and composite reliability (CR) were used to measure validity and reliability. The loadings of all the items must be more than 0.5, the average variance extracted (AVE) should be a minimum of 0.5, while the composite reliability (CR) needs to be above 0.7 for convergent validity to be attained^84,85^. As shown in Table 5, all the AVE were more significant than 0.5, and the CR were greater than 0.7. However, some loadings were lower than 0.7. The measurement was shown to have convergent validity and reliability because the AVE and CR met the specified cut-off values^86^.

**Table 5:** Measurement model for constructs.

Variable	ITEM	Loading	CR	AVE
Development culture	DC1	0.745	0.915	0.643
	DC2	0.845		
	DC3	0.860		
	DC4	0.876		
	DC5	0.754		
	DC6	0.715		
Design strategies	DS3	0.729	0.881	0.517
	DS4	0.546		
	DS5	0.823		
	DS6	0.789		
	DS7	0.645		
	DS8	0.704		
	DS9	0.761		
Economic sustainability of projects	ESCP1	0.794	0.939	0.606
	ESCP10	0.776		
	ESCP2	0.789		
	ESCP3	0.826		
	ESCP4	0.710		
	ESCP5	0.794		
	ESCP6	0.713		
	ESCP7	0.789		
	ESCP8	0.790		
	ESCP9	0.794		
Group culture	GC1	0.626	0.893	0.628
	GC2	0.832		
	GC3	0.854		
	GC4	0.820		
	GC5	0.810		
Hierarchical culture	HC1	0.887	0.805	0.516
	HC2	0.642		
	HC3	0.550		
	HC4	0.749		
Rational culture	RC1	0.834	0.880	0.552
	RC2	0.755		
	RC3	0.611		
	RC4	0.747		
	RC5	0.804		
	RC6	0.687		

DS1, DS2 and HC5 were deleted due to low loadings.

Next, discriminant validity was assessed by looking at the HTMT ratios. By empirical standards, discriminant validity refers to how distinct a construct is from others. If the ratios are less than HTMT_0.85_, it may be concluded that all measures are discriminant^87^. Also, if the upper limit of the HTMT bootstrapping value does not contain a 1, then the measurements are discriminant^88^. As shown in Table 6, all the ratios are below a cut-off value of 0.85; as such, the measures are distinct.

**Table 6:** Discriminant validity HTMT Ratio.

Variables	1	2	3	4	5	6
1. Design strategies						
2. Development culture	0.715					
3. Economic sustainability of projects	0.298	0.166				
4. Group culture	0.418	0.752	0.155			
5. Hierarchical culture	0.328	0.205	0.230	0.190		
6. Rational culture	0.311	0.704	0.130	0.735	0.279	

#### Structural model

The structural model explains the hypothesized relationship between the constructs. To check the significance level, t-statistics for all paths were evaluated using a complete bootstrapping procedure with 5000 samples, a significance level of 5 percent, and a one-tailed test^86^. The findings for direct relationships are summarized in Table 7; revealing that DS is thus associated with GC (β = 0.357, t = 7.219), DC(β = 0.637, t = 20.775), RC(β = 0.269, t = 5.344), HC(β = − 0.316, t = 7.252) and ESCP(β = 0.246, t = 2.953). Also, DC with ESCP (β = − 0.063, t = 0.662), GC with ESCP (β = 0.115, t = 1.752), HC with ESCP (β = − 0.169, t = 2.510), RC with ESCP (β = − 0.096, t = 0.859). In addition, based on the critique of^89^ that *p*-values are not a good criterion for assessing the significance of hypotheses, we employed a combination of parameters such as *p*-values, confidence intervals, and effect sizes. Table 7 shows the summary of the parameters we have used to test the hypotheses developed.

**Table 7:** Testing direct relationships.

Hypothesis	Relationship	Std. Beta	Std. Dev	t-value	*p*-value	BCI LL	BCI UL	f2	**Decision**
H2	CDS—DC	0.637	0.031	20.775	** < .001**	0.580	0.682	0.683	Supported
H5	CDS—ESCP	0.246	0.083	2.953	**0.002**	0.108	0.383	0.036	Supported
H1	CDS—GC	0.357	0.049	7.219	** < .001**	0.262	0.427	0.146	Supported
H4	CDS—HC	− 0.316	0.044	7.252	** < .001**	− 0.376	− 0.226	0.111	Not supported
H3	CDS—RC	0.269	0.050	5.344	** < .001**	0.174	0.339	0.078	Supported
H7	DC—ESCP	− 0.063	0.095	0.662	**0.254**	− 0.224	0.093	0.001	Not supported
H6	GC—ESCP	0.115	0.066	1.752	**0.040**	0.000	0.214	0.007	Supported
H9	HC—ESCP	− 0.169	0.067	2.510	**0.006**	− 0.268	− 0.046	0.027	Not supported
H8	RC—ESCP	− 0.096	0.112	0.859	**0.195**	− 0.288	0.078	0.005	Not supported

Significant values are in [bold].

**Figure 2:**
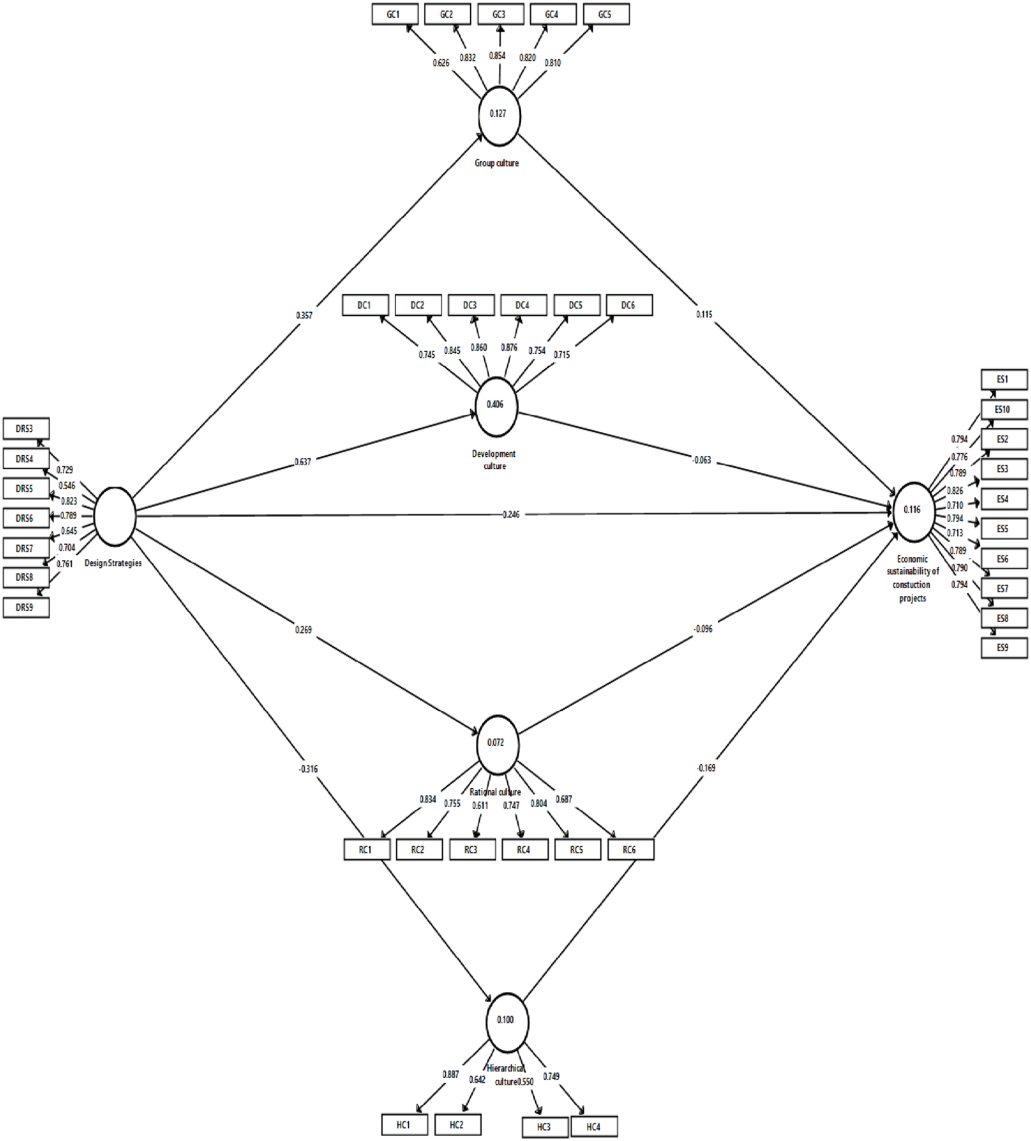
Outer loading, R2 and path coefficients.

R^2^ was analyzed to examine the amount of variance in the endogenous constructs explained by exogenous constructs^81^. The effect range is from 0 to 1, which assumes that the higher the value, the higher the predictive accuracy level^86^. This research used the rule of thumb developed, where 0.26 means substantial predictive accuracy, 0.13 means moderate predictive accuracy, and 0.02 means weak predictive accuracy. The R^2^ values for GC, DC, RC, HC and ESCP are 0.127 (moderate), 0.406 (substantial), 0.072 (weak), 0.100 (weak) and 0.116 (weak), respectively (Refer to Fig. 2). Thus, the highest predictive accuracy of 41% of CDS can be explained by DC. To measure the effect size (f^2^), the value of 0.02 is small, 0.15 is medium, and 0.35 is large^87^. As observed in Table, DC has no effect on ESCP(f^2^ = 0.001), CDS has a negligible impact on ESCP (f^2^ = 0.036), CDS has a little effect on RC(f^2^ = 0.078) and HC(f^2^ = 0.111), HC has a negligible impact on ESCP (f^2^ = 0.027), CDS has a medium effect on GC(f^2^ = 0.146), RC has no impact on ESCP(f^2^ = 0.005), GC has no effect on ESCP(f^2^ = 0.007), and CDS is having a significant impact on DC (f^2^ = 0.683). The results above indicate that hypotheses H1, H2, H3, H5 and H6 are supported based on the parameters above. Based on the same criteria, H7 and H8 are not supported. The results also show that despite not having a value of 0 between the Confidence Intervals Bias adjusted at the Upper and Lower Limits^90^, hypotheses H4 and H9 are not supported due to negative beta values. As a result, all the hypotheses for direct relationships are supported except H4, H7, H8 and H9.

#### Mediation analysis

To test the mediation hypotheses, this study followed the suggestions^81,91^ by bootstrapping the indirect effect. It can be concluded that there is significant mediation if the confidence interval does not straddle a 0. As shown in Table 8, CDS → GC → ESCP (β = 0.041, t = 1.713) and CDS → HC → ESCP (β = 0.053, t = 2.609) are significant, while CDS → DC → ESCP (β = − 0.040, t = 0.660) and CDS → RC → ESCP (β = − 0.026,t = 0.821) are not significant. Furthermore, the confidence intervals bias corrected 95% also showed intervals straddling a 0 for H11 and H12. Thus, H10 and H13 are supported, while H11 and H12 are not.

**Table 8:** Testing the Indirect Relationships.

Hypothesis	Relationship	Std. Beta	Std. Dev	t-value	*p*-values	BCI LL	BCI UL	Decision
H10	CDS → GC → ESCP	0.041	0.024	1.713	**0.043**	0.004	0.083	Supported
H13	CDS → HC → ESCP	0.053	0.020	2.609	**0.005**	0.021	0.088	Supported
H11	CDS → DC → ESCP	− 0.040	0.061	0.660	**0.255**	− 0.142	0.060	Not supported
H12	CDS → RC → ESCP	− 0.026	0.031	0.821	**0.206**	− 0.083	0.019	Not supported

Significant values are in [bold].

#### Predictive relevance ***Q***^2^

This measure is an indicator of the model’s predictive power or predictive relevance. When a PLS path model is predictively relevant, it can accurately forecast data not included in the model estimation. In the structural model, *Q*^2^ values larger than zero for a specific reflective endogenous latent variable indicate the path model’s predictive relevance for a particular dependent construct. The *Q*^2^ value is obtained using the blindfolding procedure, an iterative process that repeats until each data point has been omitted and the model re-estimated. For our model, this process was repeated 9 times. The blindfolding process is typically used on endogenous constructs with a reflecting measurement model specification and single-item endogenous constructs^81^. A *Q*^2^ score greater than 0 indicates the model predicts a specific endogenous construct^86^. Values of 0 and lower, on the other hand, show a lack of predictive relevance.

Table 9 demonstrates that all endogenous variable items in the model had a *Q*^2^ value greater than 0, indicating that the model has predictive potential and may be used to ascertain new cases.

**Table 9:** Predictive relevance Q^2^.

Variable	SSO	SSE	Q^2^	PR presence
Development culture	2,154.000	1,611.547	0.252	YES
Economic sustainability of projects	3,590.000	3,370.944	0.061	YES
Group culture	1,795.000	1,657.283	0.077	YES
Hierarchical culture	1,436.000	1,381.176	0.038	YES
Rational culture	2,154.000	2,074.047	0.037	YES

## Discussion and conclusion

This study provides a comprehensive analysis of the interplay between Circular Design Strategies (CDS), Organizational Culture (OC), and the Economic Sustainability of Construction Projects (ESCP) within the context of the Chinese construction industry. The findings corroborate previous literature and extend the understanding of these constructs and their interrelations, offering theoretical and practical implications.

The primary goal of this study is to look at the link between circular design strategies and construction projects’ economic sustainability, using organizational culture as a mediating factor (group, development, rational and hierarchical cultures). To the authors’ knowledge, this is the first empirical study in northern China that looks at this theoretical framework.

### Theoretical contribution

#### Interplay between CDS and ESCP

The research demonstrates a significant, albeit nuanced, relationship between Circular Design Strategies and the Economic Sustainability of Construction Projects. This supports existing literature that underscores the importance of sustainable practices in enhancing the economic viability of construction projects^56,60^. However, the study goes further by quantifying the strength of this relationship, shedding light on the extent to which CDS directly influences ESCP in the Chinese construction sector.

#### Influence of Organizational Culture

The study reveals that Organizational Culture significantly mediates the relationship between CDS and ESCP. This finding aligns with the works^57,61^, highlighting the pivotal role of organizational values and practices in successfully implementing sustainable strategies. The study contributes to the literature by providing empirical evidence of the mediating role of OC, especially within the hierarchical and group culture dimensions, in the context of the Chinese construction industry.

The analysis of the relationship between CDS and ESCP in northern China based on the mediating role of the CVF of OC is the research’s critical theoretical contribution (group, development, rational and hierarchical cultures). The circularity attitude is bolstered by the widespread theme of sustainable development, which has grabbed global consciousness and execution. The relationship between CDS and ESCP is yet to be studied, as mentioned in the first section. Consequent to the research questions, objectives, and subsequent findings, the research has drawn essential conclusions.

The first research question sought to ascertain the relationship between CDS and ESCP. Based on the findings, the relationship was significant, as hypothesized, although with a small effect size (0.036). Their supported hypothesis agrees with the assertion^61^, while the small and weak effect can collaborate with the studies^25,26^who concluded that companies struggle to apply CE thoughts to construction. Another reason for the perceived weakness of the relationship might be the limited knowledge of respondents on circular practices and novel ideas such as CDS. The results for the second research question we set out to answer with H1-H4 reveal that all hypotheses except the relationship between CDS and HC are supported. This makes sense and agrees with the findings^35,92^, which reflect the hierarchical culture’s difficulty in accommodating new inventions in its stringent and highly controlled setting. In contrast, the strong relationship between CDS and DC highlights how DC incorporates innovative ideas into its fold, as concluded by^19,93^.

This study also revealed that the third research question addressed by H6-H9 was not supported except for the relationship between GC and ESCP. This contradicts previous research^94,95^ that dwelled on the positive relationship between OC and ESCP. Regarding the effect of these cultures on sustaining construction economically, the hierarchical nature of firms plays a significant role, as highlighted in the results (0.027), as against development culture, which has a minor effect on the four cultures (0.001). This further validates the result from the second research question (H1–H4). The fourth research question sought to answer the mediating effect of OC on the relationship between CDS and ES. The result supports two hypotheses, H10 and H13, while H11 and H12 are not. The result confirms that the current low state of economic sustainability in the study area can be attributed to the two most prevalent culture types in construction firms: hierarchical culture (t = 2.609) and group culture (t = 1.713).

Aside from these findings, this study contributes to closing a gap in applying CDS and CE principles in a developing country like China. Most earlier studies had centred on larger organizations in advanced countries. As a result, this study adds to the current body of knowledge by studying and investigating the link between CDS and ESCP through the lens of OC in northern China. Finally, this study contributes practically as the predictive relevance Q^2^ confirms that the model can predict new cases.

### Practical implication

#### Strategic implementation of CDS

The positive relationship between CDS and ESCP underscores the importance of integrating circular design strategies in construction projects to enhance economic sustainability. Construction firms are encouraged to adopt such methods, ensuring that resource usage is optimized and waste is minimized, thereby contributing to environmental sustainability and enhancing the economic performance of construction projects.

#### Cultural reorientation for sustainability

The mediating role of Organizational Culture in the relationship between CDS and ESCP suggests that firms should foster a culture that supports sustainability initiatives. Particularly, firms with hierarchical and group cultures, which have been shown to have a significant mediating effect, should consider reorienting their cultural values to be more conducive to adopting circular design strategies. This may involve training programs, revising policy frameworks, and incentivizing sustainable practices among employees.

This paper also presents the Chinese construction industry with practical implications besides the theoretical contributions. The research conceptual framework and the output model can be used as a guide in the construction industry to assist contracting and consulting firms in understanding the impact of their respective organizational cultures in applying circular design ideologies for attaining the economic sustainability of their projects. Previous literature has highlighted these firms’ reluctance to engage these ideologies^25,26^, citing their organizational disposition as a probable reason for this shortcoming^12,27^. Using the model from this study, Partners, Administrators, Project managers and technical staff of these firms will be able to positively reposition their organizations to effectively adopt the appropriate strategy for attaining the much-required economic output for their projects. Administrators should strive to improve their knowledge of sustainable and circular practices to strengthen the relationship between CDS and ESCP.

Consequent to discovering the insignificant relationship between OC and ESCP in the study, firm administrators must spearhead an organizational paradigm shift from the current stringent culture to a more flexible and innovative culture to achieve the desired objective. This could be done simultaneously with investments in innovations, sustainable skills, and technology acquisitions to increase the economic performance of construction projects. When this is achieved, the state of the study variables, which HC and GC mediate, will improve and give way for the more flexible and innovative culture (DC) to mediate the relationship, thereby attaining ESCP in China.

### Limitations of the study

Even though this study contributes to theory and practice, it has some limitations. To begin with, data collection is solely based on self-report questionnaires. Even though some scholars have questioned this method, it was deemed necessary due to the difficulty in independently assessing each construct. Furthermore, a comprehensive collinearity test was performed, and the VIF values demonstrated that CMV was not a concern in this study. Additionally, this study used cross-sectional data, which makes it impossible to assess ESCP before and after companies adopted the circular design concept. Hence, a longitudinal study is recommended, where more detailed findings can be imparted after adjustments are made. Also, this study was conducted entirely in China, emphasizing the country’s building industry in the north. Consequently, the conclusions cannot be applied to less developed or more developed countries or other sectors within the same country. Despite these limitations, the findings offer fresh insights into CDS and ESCP in the Chinese construction industry based on the mediating role of OC.

### Directions for future research

Considering the limitations encountered for this study, it can be taken up and extended in other directions. Other circular economy processes apart from design (circular construction, material sustainability, etc.) can also be investigated to ascertain how OC or other organizational attributes can mediate or moderate their relationship with sustainability. Future research can also replicate this approach for different industries in the study area. It would also be interesting to see how these constructs relate to other dimensions of sustainability, both environmental and social.

Apart from that, future research could employ a longitudinal design to assess the impact of CDS and changes in OC on ESCP over time. Expanding the study to include other regions or industries could provide a more comprehensive understanding of these relationships.

### Conclusion

This study meticulously explored the intricate relationship between Circular Design Strategies (CDS), Organizational Culture (OC), and the Economic Sustainability of Construction Projects (ESCP) within the Chinese construction industry. It aimed to understand the direct impact of circular design strategies on economic sustainability and how the fabric of organizational culture mediates this relationship. The findings unearthed the nuanced yet impactful nature of these interrelationships, offering insights into the potential of CDS in driving economic sustainability when effectively integrated with a supportive organizational culture.

#### Summary and impactful nature of results

The research confirmed that CDS positively influences ESCP, albeit the strength of this influence varies, underscoring the importance of sustainable design practices in enhancing the economic viability of construction projects. More profoundly, the study revealed that OC significantly mediates this relationship, particularly highlighting the roles of hierarchical and group cultures. These insights are crucial, pointing to the fact that organisational culture must be aligned for circular design strategies to be effectively translated into economic sustainability. This alignment ensures that sustainable practices are adopted and deeply embedded within the firm’s operations and values.

#### Consequential effects

The consequential effects of these findings are multifaceted. This study highlights the need for construction firms to foster a culture that supports innovation and sustainability. It calls for a strategic reorientation towards practices that drive economic benefits and contribute to a sustainable construction industry. For policymakers and stakeholders, the study underscores the importance of promoting policies and frameworks that encourage the adoption of circular design strategies and foster organizational cultures conducive to sustainability.

#### Limitations and directions for future research

While the study offers valuable insights, it acknowledges certain limitations. The use of cross-sectional data limits the ability to observe the evolution of these relationships over time. Additionally, the focus on the Chinese construction industry, while offering in-depth insights, may limit the generalizability of the findings to other contexts.

Future research should consider longitudinal studies to capture the dynamics of these relationships over time. Expanding the geographical scope to include other regions or conducting comparative studies between different construction industries could provide a broader understanding of these relationships. Additionally, future studies might explore the elements within organizational culture that most effectively support the adoption and implementation of circular design strategies.

#### Final thoughts

In conclusion, this study emphasizes the critical role of circular design strategies and organizational culture in driving the economic sustainability of construction projects. It offers a nuanced understanding of how these elements interplay within the Chinese construction industry, providing a foundation for future research and practice to foster a more sustainable construction industry. The findings serve as a call to action for firms and policymakers alike to champion practices and cultures that drive economic growth and contribute to a more sustainable and resilient construction industry.

In a nutshell, the main contribution of the present research is its proof concerning the positive effect of CDS on ESCP and the discovery of adverse effects of OC on ESCP in the Chinese construction industry. As a mediator, HC weakens the relationship between CDS and ESCP. This signals that firms need to develop and switch to a more innovative culture like the DC to drive circular practices and eventually contribute to the economic sustainability of construction projects in China.

## Data Availability

The data retrieved from https://libguides.library.cityu.edu.hk/busChina/comp-dir during the current study are available from the corresponding author upon reasonable request.

## Abbreviations

CDS: Circular design strategies
ESCP: Economic sustainability of construction projects
OC: Organisational culture
CE: Circular economy
PLS-SEM: Partial least squares-structural equation modeling
HC: Hierarchical culture
GC: Group culture
UKGBC: UK green building council
ES: Economic sustainability
EoL: End-of-life
CVF: Competing values framework
RQ: Research question
AVE: Average variance extracted
